# Tetra­aqua­bis[(1-ammonio-1-phosphono­ethyl)phospho­nato]zinc(II) tetra­hydrate

**DOI:** 10.1107/S1600536809010599

**Published:** 2009-03-28

**Authors:** A. Dudko, V. Bon, A. Kozachkova, V. Pekhnyo

**Affiliations:** a Institute of General and Inorganic Chemistry, NAS Ukraine, Kyiv, prosp. Palladina 32/34, 03680 Ukraine

## Abstract

The title compound, [Zn(C_2_H_8_NO_6_P_2_)_2_(H_2_O)_4_]·4H_2_O, was synthesized by the reaction of ZnCl_2_ with 1-amino­ethane-1,1-diyldiphospho­nic acid in aqueous solution. The asymmetric unit contains one-half of the complex and two water mol­ecules of solvation. The Zn atom occupies a special position on an inversion centre. This results in a slightly distorted octa­hedral coordination environment, which consists of the O atoms from two phospho­nic acids and four water mol­ecules. The crystal structure displays N—H⋯O and O—H⋯O hydrogen bonding, which creates a three-dimensional network.

## Related literature

Diphospho­nic acids are efficient drugs for the prevention of calcification and the inhibition of bone resorption, see: Matczak-Jon & Videnova-Adrabinska (2005 ▶). Diphospho­nic acids and their metal complexes are used in the treatment of Pagets disease, osteoporosis and tumoral osteolysis, see: Szabo *et al.* (2002 ▶). For related structures, see: Li *et al.* (2006 ▶, 2007 ▶); Lin *et al.* (2007 ▶).
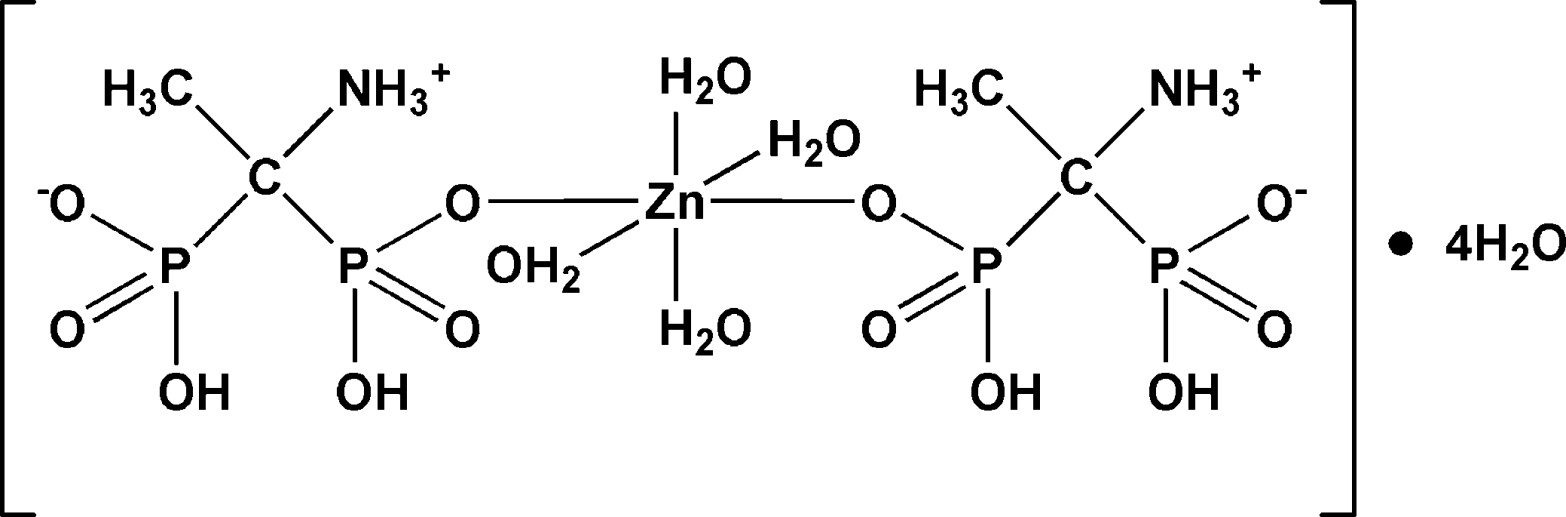


## Experimental

### 

#### Crystal data

[Zn(C_2_H_8_NO_6_P_2_)_2_(H_2_O)_4_]·4H_2_O
*M* *_r_* = 617.57
Triclinic, 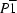

*a* = 5.6712 (4) Å
*b* = 9.3279 (6) Å
*c* = 10.7009 (7) Å
α = 96.440 (3)°
β = 90.788 (3)°
γ = 102.080 (3)°
*V* = 549.65 (6) Å^3^
*Z* = 1
Mo *K*α radiation
μ = 1.50 mm^−1^
*T* = 173 K
0.36 × 0.10 × 0.04 mm

#### Data collection

Bruker APEXII CCD diffractometer
Absorption correction: numerical (*SADABS*; Bruker, 2005 ▶) *T*_min_ = 0.612, *T*_max_ = 0.945
8897 measured reflections
2244 independent reflections
1747 reflections with *I* > 2σ(*I*)
*R*_int_ = 0.058

#### Refinement

*R*[*F* ^2^ > 2σ(*F* ^2^)] = 0.037
*wR*(*F* ^2^) = 0.081
*S* = 1.00
2244 reflections
182 parameters
1 restraint
H atoms treated by a mixture of independent and constrained refinement
Δρ_max_ = 0.39 e Å^−3^
Δρ_min_ = −0.48 e Å^−3^

### 

Data collection: *APEX2* (Bruker, 2005 ▶); cell refinement: *SAINT* (Bruker, 2005 ▶); data reduction: *SAINT*; program(s) used to solve structure: *SHELXS97* (Sheldrick, 2008 ▶); program(s) used to refine structure: *SHELXL97* (Sheldrick, 2008 ▶); molecular graphics: *SHELXTL* (Sheldrick, 2008 ▶); software used to prepare material for publication: *publCIF* (Westrip, 2009 ▶).

## Supplementary Material

Crystal structure: contains datablocks I, global. DOI: 10.1107/S1600536809010599/fj2201sup1.cif

Structure factors: contains datablocks I. DOI: 10.1107/S1600536809010599/fj2201Isup2.hkl

Additional supplementary materials:  crystallographic information; 3D view; checkCIF report

## Figures and Tables

**Table 1:** Hydrogen-bond geometry (Å, °)

*D*—H⋯*A*	*D*—H	H⋯*A*	*D*⋯*A*	*D*—H⋯*A*
N1—H1*A*⋯O6^i^	0.93 (4)	1.96 (4)	2.796 (4)	150 (3)
N1—H1*B*⋯O10	0.85 (4)	1.99 (4)	2.827 (4)	168 (3)
N1—H1*C*⋯O3^i^	0.90 (4)	2.01 (4)	2.851 (3)	153 (3)
O2—H2*O*⋯O3^ii^	0.78 (3)	1.76 (3)	2.536 (3)	172 (4)
O5—H5*O*⋯O6^iii^	0.793 (18)	1.726 (19)	2.519 (3)	177 (4)
O7—H71⋯O8^iv^	0.84 (4)	2.05 (4)	2.826 (3)	155 (3)
O7—H72⋯O10	0.76 (4)	2.00 (4)	2.748 (3)	168 (4)
O8—H81⋯O2	0.82 (4)	1.97 (4)	2.772 (3)	163 (3)
O8—H82⋯O9	0.86 (4)	1.79 (4)	2.646 (3)	174 (3)
O9—H91⋯O5^v^	0.87 (4)	1.94 (4)	2.810 (3)	172 (3)
O9—H92⋯O4^vi^	0.83 (4)	1.91 (4)	2.715 (3)	165 (4)
O10—H101⋯O4^vii^	0.85 (4)	1.90 (4)	2.744 (3)	175 (3)
O10—H102⋯O9^iv^	0.80 (4)	1.96 (4)	2.741 (3)	167 (4)

Symmetry codes: (i) 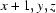; (ii) 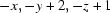; (iii) 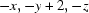; (iv) 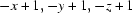; (v) 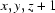; (vi) 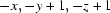; (vii) 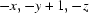.

**Fig. 1.**
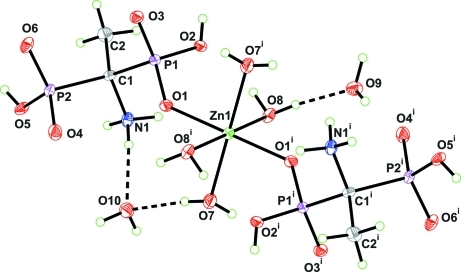
The title compound showing 50% probability displacement ellipsoids for the non-hydrogen atoms [Symmetry code: (i) -x, 1 - y, 1 - z].

**Fig. 2.**
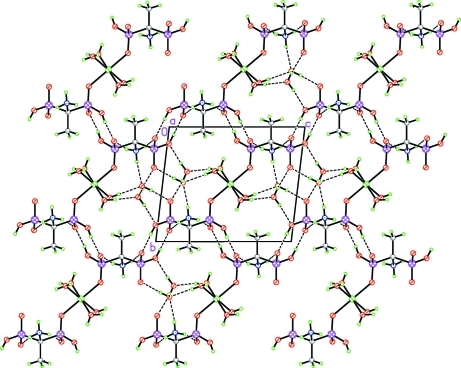
Crystal packing of title compound, projection along a axis. Dashed lines indicate hydrogen bonds.

## supplementary crystallographic information

### Comment

Organic diphosphonic acids are potentially very powerful chelating agents used in metal extractions and are tested by the pharmaceutical industry for use as efficient drugs preventing calcification and inhibiting bone resorption (Matczak-Jon *et al.*, 2005). Diphosphonic acids and their metal complexes are used in the treatment of Pagets disease, osteoporosis and tumoral osteolysis (Szabo *et al.*, 2002). The asymmetric unit of title compound contains one-half of the formula unit (Fig.1); Zn atom occupy special position at the inversion centre and creates a slightly distorted octahedral coordination environment, which consist of two phosphonic and four aqueous oxygen atoms. The coordinated diphosphonic acids residue exist as zwitterions with positive charge on NH_3_ group and negative on the oxygen atom of the non-coordinated phosphonic group. The crystal structure displays N—H···O and O—H···O hydrogen bonding, which creates a three-dimensional network (Table 1, Fig.2).

### Experimental

10 ml of the 0.01 *M* ZnCl_2_ aqueous solution was added to the 10 ml of 0.02 *M* water solution of 1-aminoethane-1,1-diyldiphosphonic acid. Colorless crystals of title compound were obtained after 2 weeks of slow evaporation of the resulted solution.

### Refinement

H atoms bonded to N and O were located in a difference map and were freely refined  with *U*_iso_(H) = 1.2 *U*_eq_ of the carrier atom. Other H atoms which bonded to C were positioned geometrically and refined using a riding model with C—H = 0.98 Å for CH_3_ [*U*_iso_(H) = 1.5Ueq(C)].

### Crystal data

**Table:**

[Zn(C_2_H_8_NO_6_P_2_)_2_(H_2_O)_4_]·4H_2_O	*Z* = 1
*M**_r_* = 617.57	*F*(000) = 320
Triclinic, *P*1	*D*_x_ = 1.866 Mg m^−^^3^
Hall symbol: -P 1	Mo *K*α radiation, λ = 0.71073 Å
*a* = 5.6712 (4) Å	Cell parameters from 2105 reflections
*b* = 9.3279 (6) Å	θ = 2.3–25.9°
*c* = 10.7009 (7) Å	µ = 1.50 mm^−^^1^
α = 96.440 (3)°	*T* = 173 K
β = 90.788 (3)°	Block, colourless
γ = 102.080 (3)°	0.36 × 0.10 × 0.04 mm
*V* = 549.65 (6) Å^3^	

### Data collection

**Table:**

Bruker APEXII CCD diffractometer	2244 independent reflections
Radiation source: fine-focus sealed tube	1747 reflections with *I* > 2σ(*I*)
graphite	*R*_int_ = 0.058
Detector resolution: 8.26 pixels mm^-1^	θ_max_ = 26.4°, θ_min_ = 2.3°
φ and ω scans	*h* = −7→7
Absorption correction: numerical (*SADABS*; Bruker, 2005)	*k* = −11→10
*T*_min_ = 0.612, *T*_max_ = 0.945	*l* = −13→13
8897 measured reflections	

### Refinement

**Table:**

Refinement on *F*^2^	Primary atom site location: structure-invariant direct methods
Least-squares matrix: full	Secondary atom site location: difference Fourier map
*R*[*F*^2^ > 2σ(*F*^2^)] = 0.037	Hydrogen site location: inferred from neighbouring sites
*wR*(*F*^2^) = 0.081	H atoms treated by a mixture of independent and constrained refinement
*S* = 1.00	*w* = 1/[σ^2^(*F*_o_^2^) + (0.0395*P*)^2^] where *P* = (*F*_o_^2^ + 2*F*_c_^2^)/3
2244 reflections	(Δ/σ)_max_ < 0.001
182 parameters	Δρ_max_ = 0.39 e Å^−^^3^
1 restraint	Δρ_min_ = −0.47 e Å^−^^3^

### Special details

**Table:**

Geometry. All e.s.d.'s (except the e.s.d. in the dihedral angle between two l.s. planes) are estimated using the full covariance matrix. The cell e.s.d.'s are taken into account individually in the estimation of e.s.d.'s in distances, angles and torsion angles; correlations between e.s.d.'s in cell parameters are only used when they are defined by crystal symmetry. An approximate (isotropic) treatment of cell e.s.d.'s is used for estimating e.s.d.'s involving l.s. planes.
Refinement. Refinement of *F*^2^ against ALL reflections. The weighted *R*-factor *wR* and goodness of fit *S* are based on *F*^2^, conventional *R*-factors *R* are based on *F*, with *F* set to zero for negative *F*^2^. The threshold expression of *F*^2^ > σ(*F*^2^) is used only for calculating *R*-factors(gt) *etc*. and is not relevant to the choice of reflections for refinement. *R*-factors based on *F*^2^ are statistically about twice as large as those based on *F*, and *R*- factors based on ALL data will be even larger.

### Fractional atomic coordinates and isotropic or equivalent isotropic displacement parameters (Å^2^)

**Table:**

	*x*	*y*	*z*	*U*_iso_*/*U*_eq_	
Zn1	0.0000	0.5000	0.5000	0.01171 (16)	
P1	0.02405 (14)	0.80873 (8)	0.37915 (7)	0.01033 (19)	
P2	−0.00669 (14)	0.81048 (9)	0.09143 (7)	0.0116 (2)	
C1	0.1808 (5)	0.8672 (3)	0.2381 (3)	0.0109 (6)	
C2	0.2791 (6)	1.0348 (3)	0.2544 (3)	0.0176 (7)	
H2A	0.3983	1.0614	0.3248	0.026*	
H2B	0.1461	1.0855	0.2716	0.026*	
H2C	0.3557	1.0644	0.1770	0.026*	
N1	0.3905 (5)	0.7921 (3)	0.2240 (3)	0.0133 (6)	
H1A	0.483 (6)	0.830 (4)	0.160 (3)	0.020*	
H1B	0.349 (6)	0.699 (4)	0.208 (3)	0.020*	
H1C	0.485 (6)	0.807 (4)	0.295 (3)	0.020*	
O1	−0.0090 (4)	0.6449 (2)	0.37040 (19)	0.0135 (5)	
O2	0.2165 (4)	0.8783 (2)	0.4886 (2)	0.0143 (5)	
H2O	0.206 (6)	0.956 (4)	0.519 (3)	0.017*	
O3	−0.1966 (4)	0.8741 (2)	0.39210 (19)	0.0139 (5)	
O4	−0.0857 (4)	0.6470 (2)	0.0759 (2)	0.0181 (5)	
O5	0.1793 (4)	0.8548 (2)	−0.0123 (2)	0.0148 (5)	
H5O	0.182 (6)	0.931 (3)	−0.039 (3)	0.018*	
O6	−0.1990 (4)	0.8997 (2)	0.0930 (2)	0.0156 (5)	
O7	0.1908 (4)	0.3844 (3)	0.3764 (2)	0.0183 (5)	
H71	0.319 (7)	0.371 (4)	0.408 (3)	0.022*	
H72	0.204 (7)	0.413 (4)	0.312 (4)	0.022*	
O8	0.3234 (4)	0.6366 (3)	0.5883 (2)	0.0152 (5)	
H81	0.311 (6)	0.719 (4)	0.571 (3)	0.018*	
H82	0.300 (6)	0.633 (4)	0.667 (4)	0.018*	
O9	0.2666 (4)	0.6106 (3)	0.8304 (2)	0.0181 (5)	
H91	0.228 (6)	0.681 (4)	0.882 (3)	0.022*	
H92	0.189 (6)	0.532 (4)	0.852 (3)	0.022*	
O10	0.3107 (4)	0.4849 (3)	0.1490 (2)	0.0187 (5)	
H101	0.236 (6)	0.448 (4)	0.079 (4)	0.022*	
H102	0.437 (7)	0.460 (4)	0.143 (3)	0.022*	

### Atomic displacement parameters (Å^2^)

**Table:**

	*U*^11^	*U*^22^	*U*^33^	*U*^12^	*U*^13^	*U*^23^
Zn1	0.0117 (3)	0.0109 (3)	0.0129 (3)	0.0029 (2)	0.0003 (2)	0.0021 (2)
P1	0.0105 (4)	0.0103 (4)	0.0105 (4)	0.0031 (3)	0.0003 (3)	0.0009 (3)
P2	0.0115 (4)	0.0125 (4)	0.0116 (4)	0.0031 (3)	−0.0009 (3)	0.0035 (3)
C1	0.0096 (15)	0.0088 (15)	0.0146 (16)	0.0030 (12)	0.0003 (12)	0.0007 (12)
C2	0.0208 (18)	0.0114 (16)	0.0183 (18)	−0.0021 (13)	0.0001 (14)	0.0017 (13)
N1	0.0105 (14)	0.0155 (15)	0.0134 (15)	0.0010 (12)	−0.0017 (11)	0.0032 (12)
O1	0.0179 (12)	0.0093 (11)	0.0133 (12)	0.0028 (9)	0.0007 (9)	0.0011 (8)
O2	0.0164 (12)	0.0141 (12)	0.0129 (12)	0.0067 (10)	−0.0032 (9)	−0.0032 (9)
O3	0.0118 (11)	0.0150 (12)	0.0154 (12)	0.0049 (9)	−0.0013 (9)	−0.0004 (9)
O4	0.0224 (13)	0.0143 (12)	0.0158 (12)	0.0001 (9)	−0.0047 (10)	0.0013 (9)
O5	0.0187 (12)	0.0118 (12)	0.0167 (12)	0.0061 (10)	0.0061 (9)	0.0080 (9)
O6	0.0113 (11)	0.0206 (12)	0.0178 (12)	0.0060 (9)	0.0024 (9)	0.0092 (9)
O7	0.0186 (13)	0.0225 (13)	0.0162 (13)	0.0090 (10)	−0.0004 (11)	0.0043 (10)
O8	0.0146 (12)	0.0139 (12)	0.0181 (13)	0.0034 (10)	−0.0017 (10)	0.0054 (10)
O9	0.0201 (13)	0.0148 (12)	0.0199 (13)	0.0042 (10)	0.0041 (10)	0.0025 (10)
O10	0.0152 (13)	0.0239 (13)	0.0176 (13)	0.0072 (11)	−0.0022 (10)	−0.0004 (10)

### Geometric parameters (Å, °)

**Table:**

Zn1—O1	2.050 (2)	C2—H2A	0.9800
Zn1—O1^i^	2.050 (2)	C2—H2B	0.9800
Zn1—O7^i^	2.071 (2)	C2—H2C	0.9800
Zn1—O7	2.071 (2)	N1—H1A	0.93 (4)
Zn1—O8^i^	2.141 (2)	N1—H1B	0.85 (4)
Zn1—O8	2.141 (2)	N1—H1C	0.90 (4)
P1—O1	1.492 (2)	O2—H2O	0.78 (3)
P1—O3	1.504 (2)	O5—H5O	0.793 (18)
P1—O2	1.575 (2)	O7—H71	0.84 (4)
P1—C1	1.839 (3)	O7—H72	0.76 (4)
P2—O4	1.486 (2)	O8—H81	0.82 (4)
P2—O6	1.503 (2)	O8—H82	0.86 (4)
P2—O5	1.571 (2)	O9—H91	0.87 (4)
P2—C1	1.846 (3)	O9—H92	0.83 (4)
C1—N1	1.502 (4)	O10—H101	0.85 (4)
C1—C2	1.535 (4)	O10—H102	0.80 (4)
			
O1—Zn1—O1^i^	179.999 (1)	N1—C1—P1	107.2 (2)
O1—Zn1—O7^i^	90.77 (9)	C2—C1—P1	110.6 (2)
O1^i^—Zn1—O7^i^	89.23 (9)	N1—C1—P2	106.55 (19)
O1—Zn1—O7	89.23 (9)	C2—C1—P2	110.3 (2)
O1^i^—Zn1—O7	90.77 (9)	P1—C1—P2	113.58 (16)
O7^i^—Zn1—O7	180.00 (11)	C1—C2—H2A	109.5
O1—Zn1—O8^i^	88.74 (9)	C1—C2—H2B	109.5
O1^i^—Zn1—O8^i^	91.26 (9)	H2A—C2—H2B	109.5
O7^i^—Zn1—O8^i^	92.46 (9)	C1—C2—H2C	109.5
O7—Zn1—O8^i^	87.54 (9)	H2A—C2—H2C	109.5
O1—Zn1—O8	91.27 (9)	H2B—C2—H2C	109.5
O1^i^—Zn1—O8	88.73 (9)	C1—N1—H1A	108 (2)
O7^i^—Zn1—O8	87.54 (9)	C1—N1—H1B	114 (2)
O7—Zn1—O8	92.46 (9)	H1A—N1—H1B	110 (3)
O8^i^—Zn1—O8	180.0	C1—N1—H1C	113 (2)
O1—P1—O3	118.11 (12)	H1A—N1—H1C	108 (3)
O1—P1—O2	107.82 (12)	H1B—N1—H1C	104 (3)
O3—P1—O2	111.01 (12)	P1—O1—Zn1	133.80 (13)
O1—P1—C1	107.13 (13)	P1—O2—H2O	115 (3)
O3—P1—C1	108.84 (13)	P2—O5—H5O	118 (3)
O2—P1—C1	102.79 (13)	Zn1—O7—H71	113 (2)
O4—P2—O6	117.67 (13)	Zn1—O7—H72	114 (3)
O4—P2—O5	108.21 (12)	H71—O7—H72	114 (4)
O6—P2—O5	110.56 (12)	Zn1—O8—H81	101 (2)
O4—P2—C1	108.34 (13)	Zn1—O8—H82	103 (2)
O6—P2—C1	108.52 (13)	H81—O8—H82	108 (3)
O5—P2—C1	102.44 (13)	H91—O9—H92	106 (3)
N1—C1—C2	108.4 (3)	H101—O10—H102	103 (3)
			
O1—P1—C1—N1	−47.7 (2)	O6—P2—C1—C2	−54.5 (2)
O3—P1—C1—N1	−176.47 (18)	O5—P2—C1—C2	62.5 (2)
O2—P1—C1—N1	65.8 (2)	O4—P2—C1—P1	−58.45 (19)
O1—P1—C1—C2	−165.7 (2)	O6—P2—C1—P1	70.38 (18)
O3—P1—C1—C2	65.6 (2)	O5—P2—C1—P1	−172.68 (15)
O2—P1—C1—C2	−52.2 (2)	O3—P1—O1—Zn1	−92.94 (19)
O1—P1—C1—P2	69.67 (18)	O2—P1—O1—Zn1	33.8 (2)
O3—P1—C1—P2	−59.08 (18)	C1—P1—O1—Zn1	143.85 (17)
O2—P1—C1—P2	−176.85 (15)	O7^i^—Zn1—O1—P1	41.88 (18)
O4—P2—C1—N1	59.3 (2)	O7—Zn1—O1—P1	−138.12 (18)
O6—P2—C1—N1	−171.88 (19)	O8^i^—Zn1—O1—P1	134.32 (18)
O5—P2—C1—N1	−54.9 (2)	O8—Zn1—O1—P1	−45.68 (18)
O4—P2—C1—C2	176.7 (2)		

Symmetry codes: (i) −*x*, −*y*+1, −*z*+1.

### Hydrogen-bond geometry (Å, °)

**Table:**

*D*—H···*A*	*D*—H	H···*A*	*D*···*A*	*D*—H···*A*
N1—H1A···O6^ii^	0.93 (4)	1.96 (4)	2.796 (4)	150 (3)
N1—H1B···O10	0.85 (4)	1.99 (4)	2.827 (4)	168 (3)
N1—H1C···O3^ii^	0.90 (4)	2.01 (4)	2.851 (3)	153 (3)
O2—H2O···O3^iii^	0.78 (3)	1.76 (3)	2.536 (3)	172 (4)
O5—H5O···O6^iv^	0.79 (2)	1.73 (2)	2.519 (3)	177 (4)
O7—H71···O8^v^	0.84 (4)	2.05 (4)	2.826 (3)	155 (3)
O7—H72···O10	0.76 (4)	2.00 (4)	2.748 (3)	168 (4)
O8—H81···O2	0.82 (4)	1.97 (4)	2.772 (3)	163 (3)
O8—H82···O9	0.86 (4)	1.79 (4)	2.646 (3)	174 (3)
O9—H91···O5^vi^	0.87 (4)	1.94 (4)	2.810 (3)	172 (3)
O9—H92···O4^i^	0.83 (4)	1.91 (4)	2.715 (3)	165 (4)
O10—H101···O4^vii^	0.85 (4)	1.90 (4)	2.744 (3)	175 (3)
O10—H102···O9^v^	0.80 (4)	1.96 (4)	2.741 (3)	167 (4)

Symmetry codes: (ii) *x*+1, *y*, *z*; (iii) −*x*, −*y*+2, −*z*+1; (iv) −*x*, −*y*+2, −*z*; (v) −*x*+1, −*y*+1, −*z*+1; (vi) *x*, *y*, *z*+1; (i) −*x*, −*y*+1, −*z*+1; (vii) −*x*, −*y*+1, −*z*.
